# Geospatial pattern of HIV seropositivity and its predictors among women in Ethiopia. A spatial and multiscale geographically weighted regression analysis

**DOI:** 10.1371/journal.pone.0306645

**Published:** 2024-07-11

**Authors:** Tegene Atamenta Kitaw, Biruk Beletew Abate, Befkad Derese Tilahun, Ribka Nigatu Haile

**Affiliations:** Department of Nursing, College of Health Science, Woldia University, Woldia, Ethiopia; University of Uyo, NIGERIA

## Abstract

**Background:**

Although promising efforts have been made so far, HIV remains a public health concern. Women in Ethiopia are disproportionately affected by HIV, accounting for a majority of new infections and AIDS-related deaths. However, the geospatial distribution of HIV among women in Ethiopia is not well understood, making it challenging to develop geographically targeted measures. Besides, to accelerate the pathway of decreasing HIV prevalence and plan geographically specific interventions, understanding the geospatial distribution of HIV seropositivity and its predictors among women plays a significant role.

**Methods:**

A spatial and multiscale geographically weighted regression analysis was conducted using the 2016 EDHS dataset, comprising 14,778 weighted samples of women in the reproductive age group. The EDHS sample underwent two-stage stratification and selection. The data were extracted between October 18 and 30, 2023. Non-spatial analysis was carried out using STATA version 17. Additionally, ArcGIS Pro and Sat Scan version 9.6 were used to visually map HIV seropositivity. Global Moran’s I was computed to evaluate the distribution of HIV seropositivity. The Getis-Ord Gi* spatial statistic was utilized to identify significant spatial clusters of cold and hot spot areas. Geographically weighted regression analysis was subsequently performed to identify significant predictors of HIV seropositivity. Significance was established at a P-value <0.05 throughout all statistical analyses.

**Results:**

HIV seropositivity among women in Ethiopia is distributed non-randomly (Global Moran’s I = 0.16, p-value <0.001 and Z-score = 7.12). Significant hotspot clustering of HIV seropositivity was found in the Addis Ababa, Harari, Dire Dawa, and Gambela region. Poor wealth index, being divorced and widowed, having more than one sexual partner, and early first sexual experience (<15 years) were found to be predictors of geographical variation of HIV seropositivity among women.

**Conclusion:**

HIV seropositivity among women in Ethiopia varies geographically. Thus, deploying additional resources in high hotspot regions is recommended. Programs should focus on improving the economic empowerment of women to prevent the from engaging in risky sexual behaviors. Furthermore, comprehensive sex education programs in schools and community settings regarding the consequences of early first sexual debut might play a role in reducing HIV seropositivity among women in Ethiopia.

## Introduction

Despite promising efforts, HIV remains a significant global public health concern. Worldwide, at the end of 2022, approximately more than 39 million people were infected. Two-thirds of the cases stemmed from Africa, with women and girls comprising 53% of the total [1]. Regional data from the World Health Organization revealed that in 2022, an estimated 3.8 million people were living with HIV in the Americas, 3.9 million in the Southeast Asian region, and 3.0 million in Europe [2]. Women and girls made up 63% of all new HIV infections in sub-Saharan Africa. In 2022, 4,000 teenage girls and young women between the ages of 15 and 24 worldwide contracted HIV each week. Sub-Saharan Africa was the site of 3100 of these illnesses [3].

There have been remarkable achievements in controlling the HIV/AIDS epidemic in Ethiopia over the past decade. However, due to the significant variation between urban and rural areas, it remains a public health issue [4].

Women living with HIV/AIDS face immense challenges, including profound physical and psychological consequences [5]. In addition, they often also endure painful and shameful lives, characterized by exclusion from family, friends, and partners. Thousands have lost their lives, and many others struggle to fully live despite the burden of disease. Stigma and discrimination further exacerbate the challenges faced by women living with HIV/AIDS [6]. The impact of HIV stigma on women is far-reaching. It also leads to rejection from friends, family, and society, resulting in feelings of uncertainty, loss, low self-esteem, fear, anxiety, depression, and even suicidal thoughts [7]. By 2022, according to the HIV Related Estimates and Projections, 380,495 females were living with HIV in Ethiopia [8]. In addition, the prevalence of HIV increased twofold among women (1.2%) compared with men (0.6%). Among women, HIV seropositivity increases with age, with a 0.4% increase among women aged 15–19 years and a 3% increase among women aged 44–44 years [9]. In resource-limited areas such as Ethiopia, HIV causes a substantial economic burden, especially among people with low socioeconomic status. The direct and indirect financial costs related to HIV are high [10].

The high incidence and prevalence of HIV in women are caused by a variety of factors. High risk sexual behaviors that are linked with HIV prevalence include early sexual experience, having multiple sexual partners, insufficient condom utilization, and intimate partner violence [11–13]. Socioeconomic and demographic factors, including the age of the woman, marriage status, educational attainment level, residence status, and poor wealth level, have also been related to HIV seropositivity [14, 15].

Ethiopia is responding to the HIV/AIDS epidemic with a comprehensive and integrated approach that includes combination HIV prevention, prevention in key and priority populations, HIV case finding and testing strategies, HIV care and treatment, TB/HIV co-infection treatment, and interventions to prevent and treat HIV-related cancers and other co-infections [16]. By 2030, Ethiopia aims to have 95% of people living with HIV know their status, 95% of people living with HIV start antiretroviral therapy, and 95% of people on antiretroviral therapy achieve viral load suppression [17].

The availability of free ART and the establishment of a national HIV governing body have played a significant role in reducing the impact of HIV [18, 19]. However, there are still substantial disparities in HIV prevalence across the country. There is a notable difference in HIV seropositivity rates between rural and urban areas, with rates ranging from 0.4% in rural areas to 2.9% in urban areas [20]. This underscores the heterogeneous burden of HIV in the country. Thus, despite some progress, there is still more to do.

Women in Ethiopia bear a disproportionate burden of HIV, with the majority of new infections and AIDS-related deaths occurring among them. Most studies in Ethiopia solely reported the prevalence of HIV across regions [21–23]. A research gap has been identified regarding the geospatial distribution of HIV among women in Ethiopia, which poses challenges for the development of targeted interventions. Moreover, factors influencing disparities in infection rates across different regions remain poorly understood. Spatial analysis provides a means to explore these disparities and uncover localized patterns that may not be discernible in aggregate data. By identifying spatially varying relationships in HIV prevalence across different geographical areas, policymakers can allocate resources more effectively and develop interventions tailored to address specific contributing factors. Understanding the geospatial distribution of HIV seropositivity and its predictors among women is crucial for accelerating progress toward ending HIV transmission and planning geographically specific interventions effectively. The findings provide valuable insights by enabling HIV/AIDS programs to target resources and interventions more effectively. This study contributes to the body of knowledge on HIV/AIDS epidemiology, particularly in the context of women in Ethiopia. Researchers can build upon these findings to explore innovative approaches for reducing HIV transmission and conducting further research. Additionally, the findings have implications for the community at large, especially for women at risk of HIV infection.

## Methods

### Study setting, study period and data source

According to forecasts from trading economics and data from recent census figures, the total population of Ethiopia was 115.0 million by 2020 [24]. This study uses the EDHS 2016 dataset. The EDHS report includes inclusive data at the country level from nine regional states and two municipal administrations. The administrative levels were divided into zones, woredas, and so forth. Spatial and multiscale geographically weighted regression analyses were conducted among women of reproductive age (15–49). The EDHS collects pertinent information mainly regarding maternity health care utilization, marriage and sexual behavior, child feeding practices, HIV status, children’s and women’s dietary conditions, and children’s and adult’s mortality [9]. The EDHS 2016 survey was conducted from January 18, 2016, to June 27, 2016. In Ethiopia, socioeconomic factors such as poverty and limited access to healthcare can increase vulnerability to HIV infection and hinder access to prevention, treatment, and support services. Cultural beliefs and norms may influence sexual behaviors, attitudes toward HIV/AIDS, and access to reproductive health services. Additionally, residence in rural or urban areas can affect access to healthcare, education, and employment opportunities, which in turn influence HIV/AIDS risk and outcomes. Family dynamics, including gender roles, power dynamics, and communication patterns, also shape HIV/AIDS risk and prevention strategies within households and communities [25, 26].

### Data collection, extraction, population and data quality assurance

The EDHS 2016 primarily uses standardized questionnaires that cover a wide range of topics related to demographics, health, and nutrition. Data collectors for EDHS 2016 surveys are typically field workers who are recruited and trained specifically for the survey. They undergo extensive training on survey procedures, questionnaire administration, ethical considerations, and data quality assurance. The EDHS 2016 survey was conducted from January 18, 2016, to June 27, 2016. The survey typically employ structured types of interviews with predetermined questionnaires. The EDHS 2016 survey is overseen by experienced supervisors who are responsible for managing the data collection teams in the field. The project protocol was submitted to the Demographic and Health Surveys (DHS) Program for review. After approval, the DHS Program granted access to the survey datasets on October 16, 2023. Data extraction was performed between October 18 and 30, 2023, to select women in the reproductive age group. The authors had no access to information that could identify individual participants. The source population was all Ethiopian women of reproductive age, and the study population was all Ethiopian women of reproductive age in the selected enumeration area. All methods were performed in accordance with the relevant guidelines and regulations. To maintain data quality, data collection tools underwent rigorous pretesting to identify any potential issues with clarity, relevance, or comprehensiveness. The data collectors received thorough training to ensure uniformity in administering the survey instruments and adhering to the protocols. Following the fieldwork, a debriefing session was conducted to gather feedback from the data collectors regarding their experiences and any challenges encountered during data collection.

### Sampling methods

**Fig 1:**
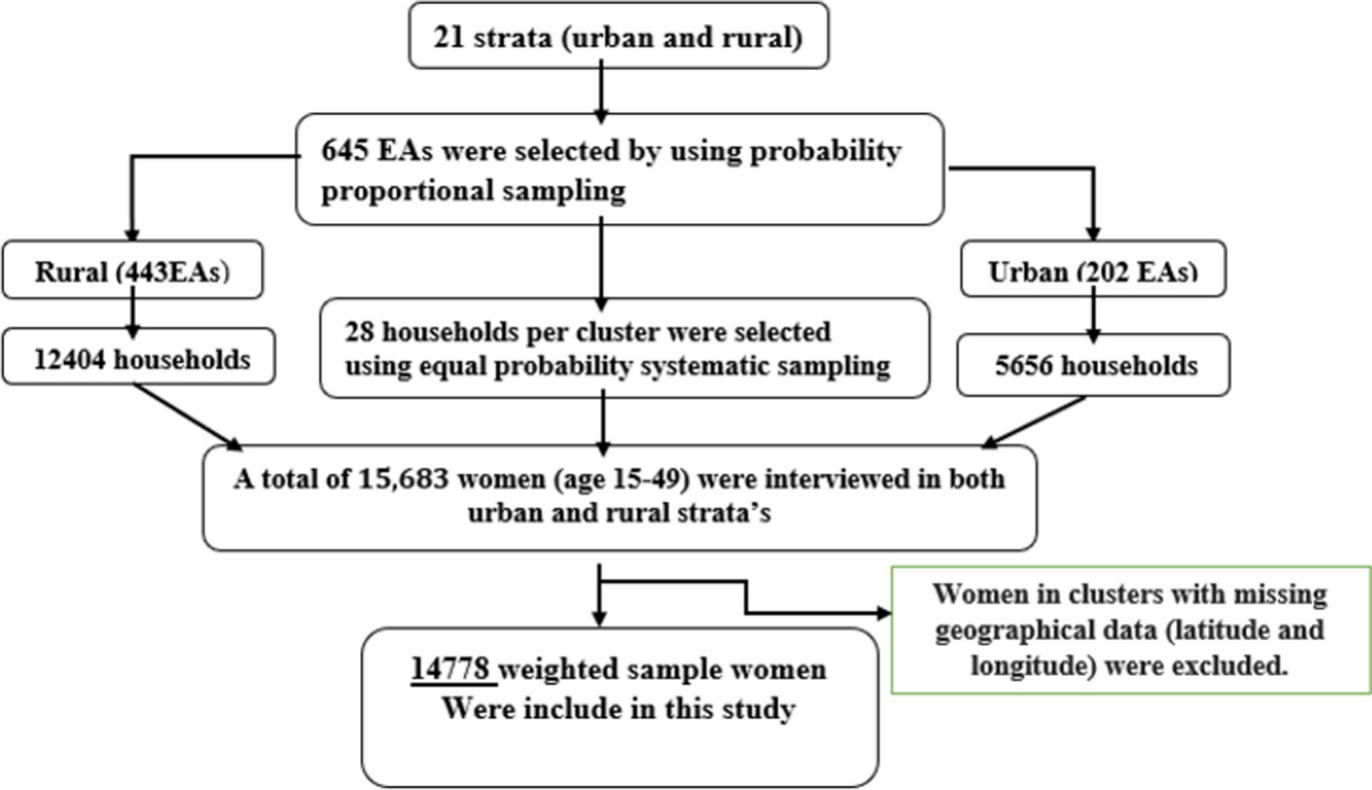
Schematic representation of the sampling procedures used to study the geospatial pattern of HIV seropositivity and its predictors among women in Ethiopia, EDHS 2016. N.B EAs = Enumeration areas.

The Ethiopia Demographic and Health Survey (EDHS) 2016 sample was selected in two stages. In the first stage, 21 sampling strata were created by stratifying each region into urban and rural areas. Then, 645 enumeration areas (202 urban and 443 rural) were selected using probability proportion sampling. In the second stage, a newly created household listing was used to select 28 households per cluster using systematic sampling with equal probability. Sample allocation was performed to ensure that survey precision was equivalent across regions [9]. In this study, a total of 14778 weighted samples of women in the reproductive age group were included. The spotlight sampling technique used in this study is shown in the figure below (**Fig 1**).

### Study variables

The outcome variable was HIV seropositivity. The explanatory variables considered for the study to determine significant predictors of the spatial distribution of HIV seropositivity were shown in the table below **(Table 1)**.

**Table 1:** List of independent variables for the assessment of the geospatial pattern of HIV seropositivity and its predictors among women in Ethiopia, EDHS 2016.

Variable	Descriptions (classification)
**Women age**	15–24, 25–34, 35–49
**Residence status**	Urban or Rural
**Region**	Larger central: Tigray, Amhara, Oromia, SNNPR
	Small peripherals: Benishangul, Gambela, Afar, Somali
	Metropolis: Harari, Addis Ababa, Dire Dawa. [27, 28]
**Mother Educational status**	No education, Primary, Secondary or higher
**Wealth index**	Poor, Middle, and Rich
**Marital status**	Never married, married, widowed, divorced
**Number of sexual partners**	One, more than one
**Ever not heard about STI**	Yes, No
**Age of first sexual experience**	<15 years, ≥15 year

### Definitions

HIV seropositivity was declared based on the 2016 EDHS HIV testing algorithm, which is based on the low prevalence HIV diagnostic algorithm recommended in the 2015 WHO guidelines [29]. All samples were first tested with an enzyme-linked immunosorbent assay (ELISA) I. If the ELISA I test result was negative, the sample was classified as HIV-negative. All samples that tested positive on ELISA I were then subjected to an ELISA II test. If both the first and second ELISA tests were positive, the sample was tested with a line immunoassay (LIA). If the LIA test was also positive, the sample is classified as HIV positive. The detailed algorithm was described in the EDHS report [30]. The outcome variable is dichotomized as “0” if the test result is negative or “1”if it is positive.

### Data processing and analysis

The data were obtained from the EDHS 2016 dataset using STATA version 17. Sorting and listing were used to find any missing values. Descriptive statistics were calculated using frequencies and percentages. The data were weighted, cleaned, edited, and recoded. STATA version 17 was used for non-spatial analysis. ArcGIS Pro and Sat Scan version 9.6 were used to map HIV seropositivity at the regional and district levels.

### Spatial autocorrelation analysis

Spatial autocorrelation with Global Moran’s I was computed to determine whether HIV seropositivity among women was randomly distributed, clustered, or dispersed. A global Moran’s I value near “0” indicates that HIV seropositivity is randomly distributed, a value near “–1” indicates dispersion, and a value close to “+1” indicates clustering. Spatial autocorrelation was indicated by at a statistically significant Moran’s I p-value less than 0.05.

### Hot spot and spatial interpolation analysis

Hot Spot Analysis was done using Getis-Ord Gi* spatial statistics to identify statistically significant spatial clusters of cold spots(low) and hot spots (high) of HIV seropositivity. Clusters with high Gi* values are statistically significant hot spots, while clusters with low Gi* values are statistically significant cold spots. Ordinary kriging interpolation was computed to estimate the values at unsampled locations from the sampled data points.

### Satscan analysis

Purely spatial analysis using the Bernoulli model was done to identify the geographical locations of statistically significant clusters for HIV seropositivity among women in Ethiopia. The likelihood ratio test statistic and the p-value were used for each potential cluster to determine whether the number of observed HIV seropositive women within the potential cluster was significantly greater than expected.

### Ordinary least squares and multiscale geographically weighted regression analysis

Ordinary least squares (OLS) regression was used to model the relationship between HIV seropositivity and a set of independent variables, using data from a sample of 620 enumeration areas. The outcome variable for the regression model was the weighted HIV prevalence in each cluster. OLS regression assumes that the relationship between each explanatory variable and the outcome variable is the same throughout the study area [31]. However, a constant relationship between the explanatory variables and the outcome variable may not always be valid. Thus, further exploration through geographically weighted regression (GWR) is crucial, because it allows the coefficients to vary across the study area.

A newly emerging geographic regression model called MGWR was used to explore the geographically varying relationships between explanatory variables. Unlike GWR, MGWR not only allows the coefficients to vary over spatially but also allows the scale to vary across different covariates. The MGWR considers distinct neighborhoods for each covariate to account for different spatial scales of the relationships between each explanatory variable and the outcome variable [32]. MGWR is a powerful tool that can be used to model complex spatial relationships between variables. The variance inflation factor was calculated to assess the existence of multicollinearity between variables. A VIF above 4 indicates that multicollinearity might exist [33]. The akaike information criterion (AIC) and adjusted R^2^ values were computed to select the appropriate model. The model with the lowest AICc and highest adjusted R^2^ was declared the best-fitting model [34]. In all the statistical analyses, P-value <0.05 indicated statistical significance.

### Ethical statements

The EDHS 2016 underwent an ethical review by the National Research Ethics Review Committee (NRERC) of the Ethiopian Ministry of Science and Technology. As detailed in the survey’s final report, participation in the survey program was voluntary, and verbal informed consent was obtained. Confidentiality was ensured by maintaining a 2 km and 5 km distance between households in urban and rural areas, respectively [30]. As stated in the EDHS HIV prevalence report, Interviewers explained the procedure and the confidentiality of the data.

### Informed consent

Informed consent was taken from each participant. If a respondent consented to HIV testing, five blood spots from the finger prick were collected on a filter paper card. For the children under the age of 18, informed consent was obtained from the parent or guardian of the child. The detailed informed consent and sample collection procedure is available in the EDHS 2016 HIV prevalence report [9].

## Results

### Basic descriptive characteristics of the study participants

A total of 14778 women in the reproductive age group participated in this study. A total of 45.5% of women were illiterate. A total of 38.7% of the study participants were in the poor wealth quantile group. The highest prevalence of HIV was found among women aged 25–49 years (0.89%) and those who lived in urban residences (1.41%) (**Table 2**).

**Table 2:** Basic descriptive characteristics of women’s in the reproductive age group in Ethiopia.

Characteristics	Categories	HIV seropositivity status	
		Negative	Positive
		Weighted	Weighted
		frequency (%)	frequency (%)
**Age**	15–24	5928(40.11%)	29(0.20%)
	25–35	4701(31.81%)	124(0.84%)
	35–49	3864(26.15%)	132(0.89%)
**Marital status**	Never married	3850(26.05%)	30(0.20%)
	Married	9134(61.81%)	133(0.90%)
	Widowed	415(2.80%)	52(0.35%)
	Divorced	1094(7.40%)	70(0.47%)
**Residence**	Urban	4587(31.04%)	208(1.41%)
	Rural	9906(67.03%)	77(0.52%)
**Educational level**	No education	6648(44.99%)	76(0.51%)
	Primary	4836(32.72%)	128(0.87%)
	Secondary and above	3009(20.36%)	81(0.55%)
**Wealth index level**	Poor	5676(38.41%)	43(0.29%)
	Meddle	1929(13.05%)	15(0.10%)
	Rich	6888(46.61%)	227(1.54%)
**Region**	Larger central	6946(47.00%)	75(0.51%)
	Small peripherals	4330(29.3%)	81(0.55%)
	Metropolis	3117(21.77%)	129(0.87%)

### Spatial and incremental autocorrelation

**Fig 2:**
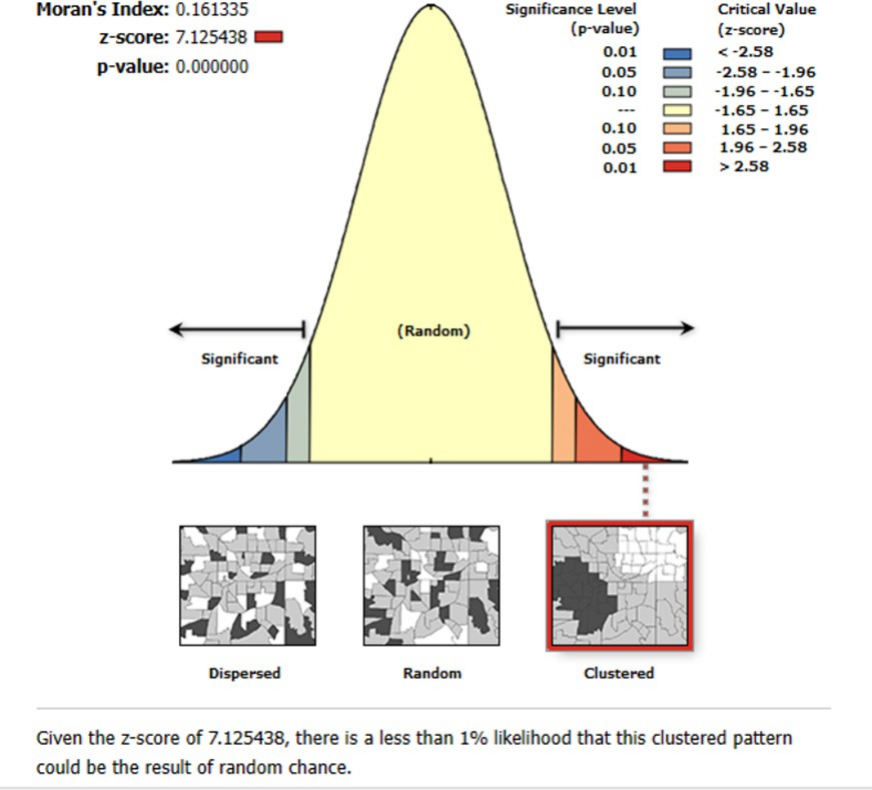
Spatial autocorrelation analysis of HIV seropositivity among women in Ethiopia.

**Fig 3:**
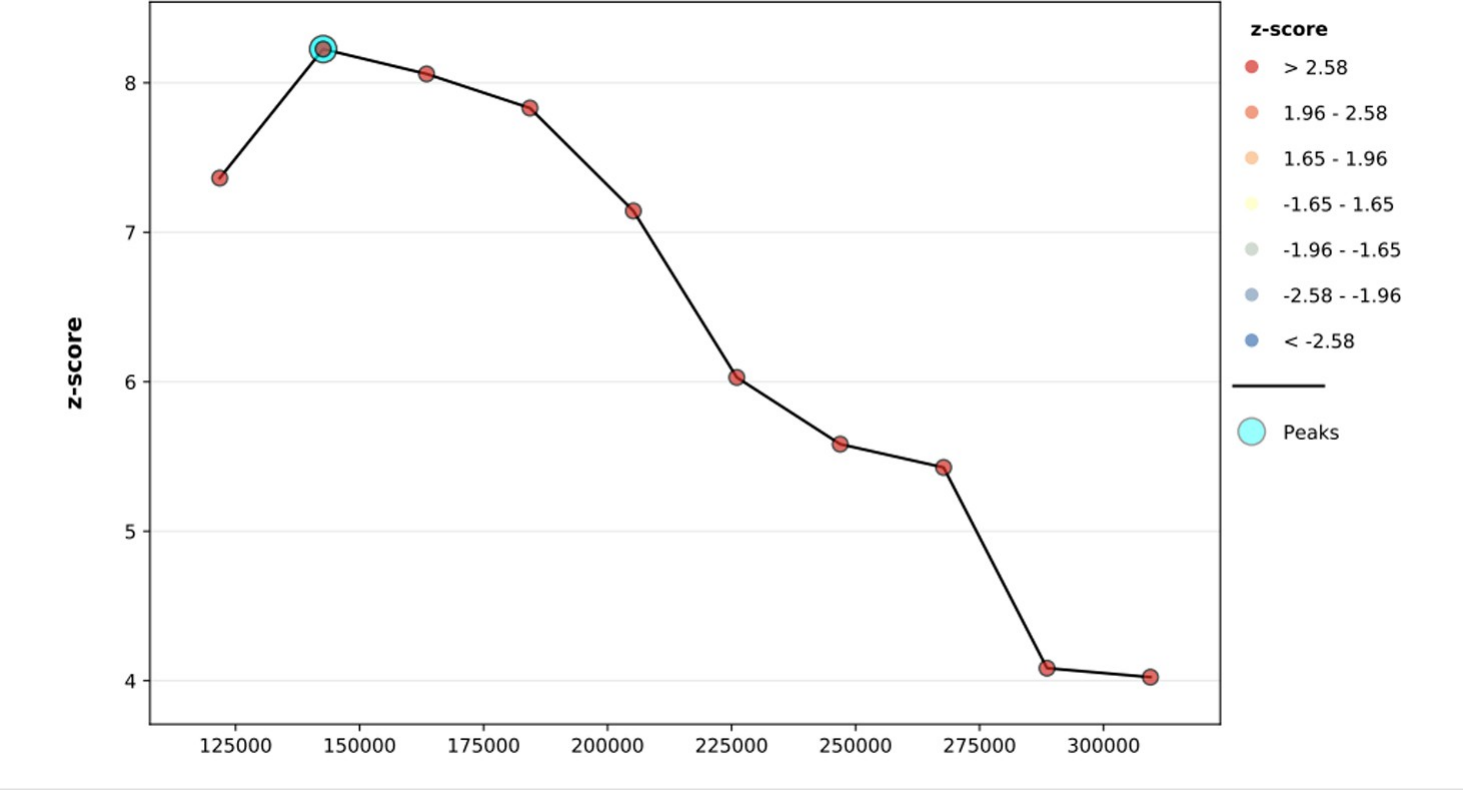
Incremental autocorrelation analysis of HIV seropositivity among women in Ethiopia.

The spatial distribution of HIV seropositivity among women in Ethiopia is clustered with global Moran’s I value of 0.16 and p-value <0.001. Thus, HIV seropositivity has a spatial dependency **(Fig 2)**. The line graph of incremental autocorrelation shows the minimum and maximum distance bands. The minimum distance at the beginning was 121801.00 meters (Z-score = 7.36, P-value<0.001), whereas the first maximum peak was 142650.16 meters (Z-score = 8.22, P-value<0.001) **(Fig 3)**.

### Hotspot and cold spot analysis

**Fig 4:**
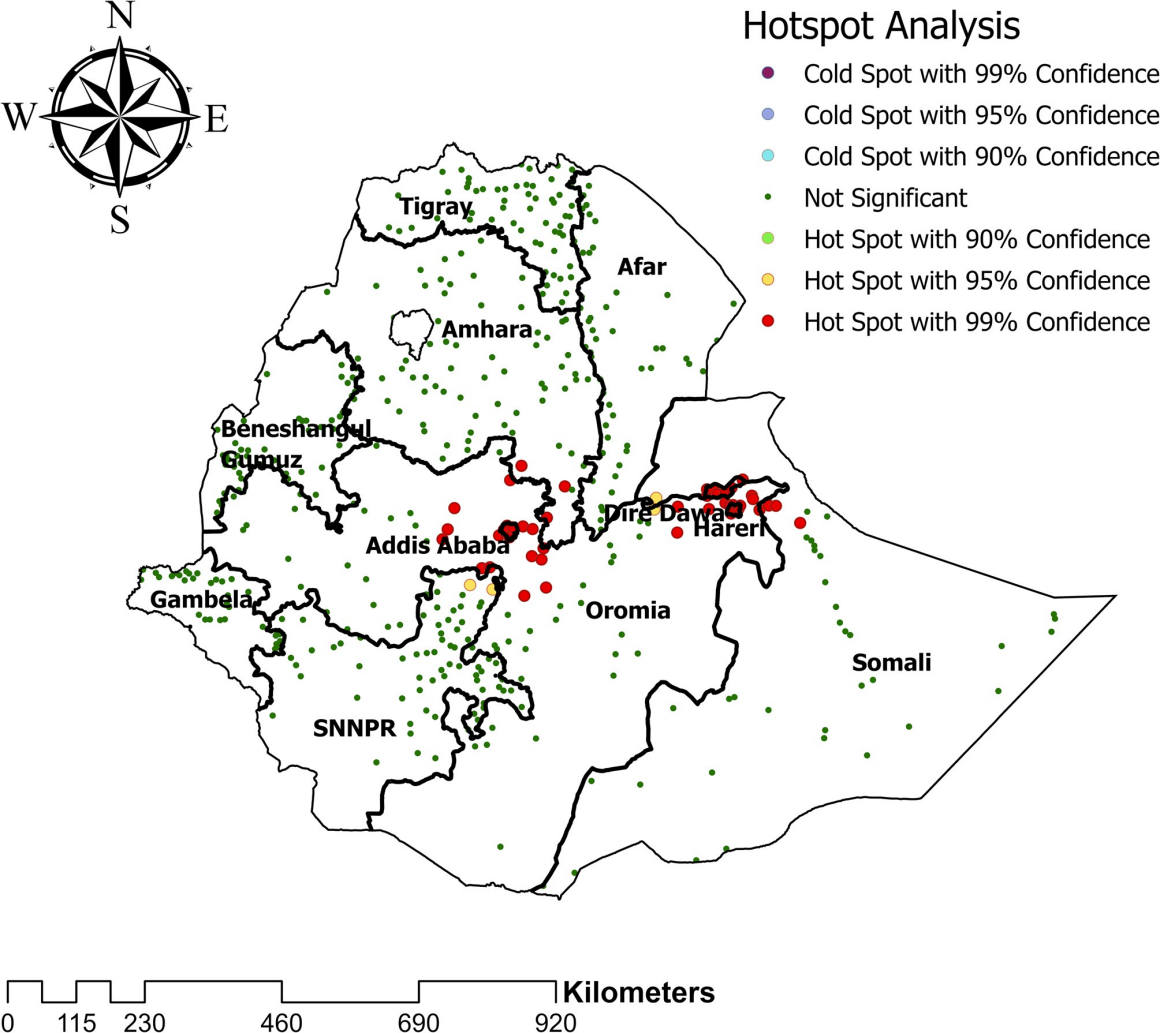
Hot spots analysis of HIV seropositivity among women in Ethiopia.

Hotspot and cold spot analyses were computed to detect areas with high and low cases of HIV seropositivity. Thus, significant hotspot clustering of HIV seropositivity was detected in Addis Ababa, Harari, and Dire Dawa **(Fig 4)**.

### Spatial interpolation

**Fig 5:**
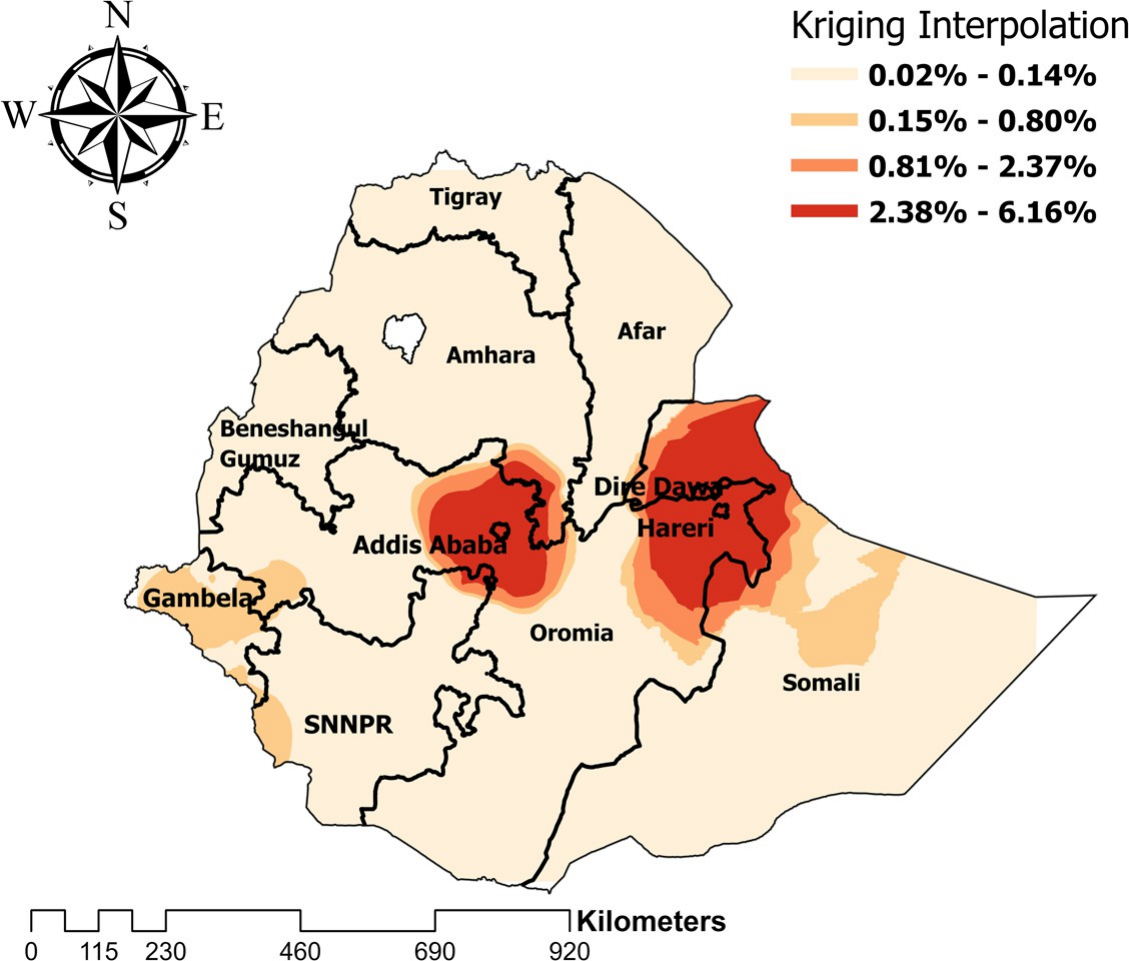
Ordinary kriging interpolation to predict HIV seropositivity prevalence among women in Ethiopia.

Ordinary kriging interpolation was computed to predict the distribution of HIV seropositivity among women in Ethiopia. Thus, the highest number of predictive HIV cases were detected in the Addis Ababa, Harari, Dire Dawa, and Gambela region. Whereas low predictive cases were found in the remaining regions of Ethiopia **(Fig 5)**.

### Satscan analysis

**Fig 6:**
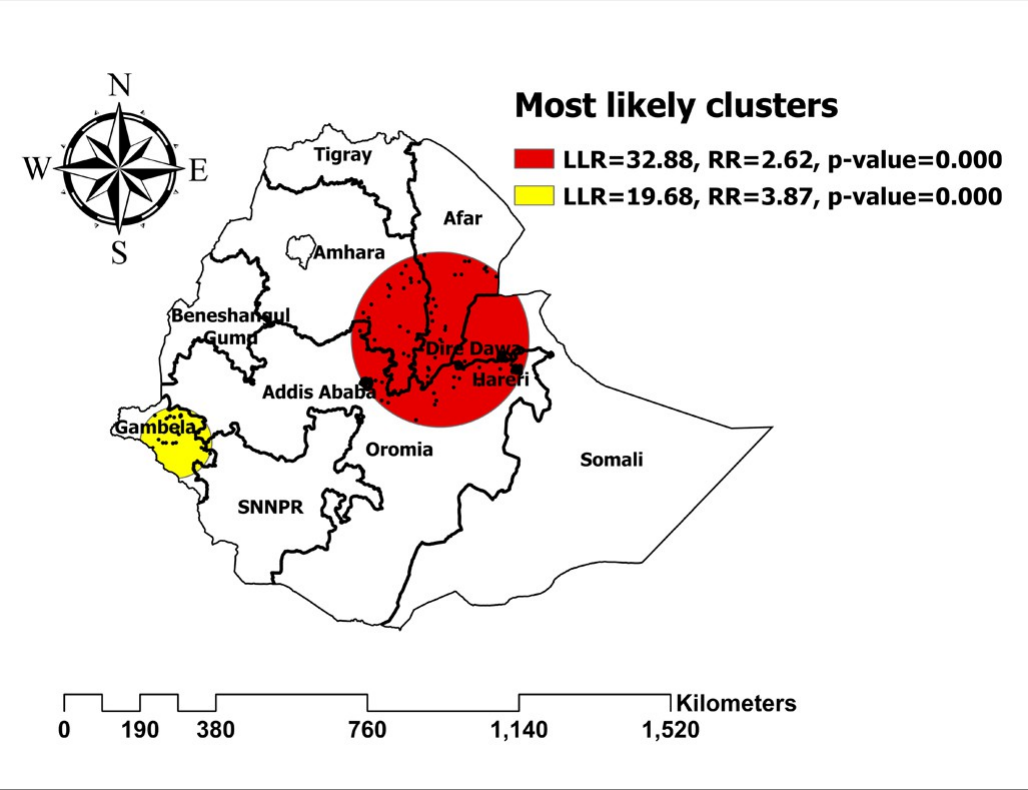
Spatial clustering of high and low rate of HIV seropositivity among women in Ethiopia.

Purely spatial analysis using the Bernoulli model was done to identify clusters with high or low HIV seropositivity. The primary significant sat scan was identified in Eastern Ethiopia (Harari, Dire Dawa) and the capital city (Addis Ababa) at 9.963904 N, 40.440496 E (radius of 218.94 km). In highly cluttered areas, there was more than two and a half times the risk for HIV seropositivity. The prevalence of HIV seropositivity was greater in the circle hole than in the outside **(Fig 6)**.

### Ordinary least squares (OLS) model results

An ordinary least squares model was computed to identify spatial determinants of HIV seropositivity. Poor wealth index, being divorced and widowed, having more than one sexual partner, and having first sexual experience at <15 years of age were found to be related to HIV seropositivity among women in Ethiopia. Multicollinearity was checked by computing the variance inflation factor (VIF). The maximum and minimum VIFs were 2.70 and 1.12, respectively. Thus, there is no significant multicollinearity between the explanatory variables. The adjusted R-squared (R^2^ = 0.081) revealed that there was 8.1% variability in HIV seropositivity. The Joint F-Statistic and Joint Wald Statistic result (P-value <0.05) shows that the model is statistically significant.

On the other hand, the Jarque-Bera Statistic result (P-value >0.05) indicated that the OLS was free from bias. Furthermore, Koenker’s (BP) Statistic was found to be statistically significant (P-value<0.05). Therefore, there is a possibility of heteroscedasticity and/or nonstationarity. Thus, the model is a good candidate for further multiscale geographically weighted regression (GWR) analysis (**Table 3**).

**Table 3:** Summary ordinary least squares (OLS) regression result.

Variables		Coefficients		Robust t-statistics	Robust probability	VIF
Intercept		3.79		6.92	0.000	‐‐‐‐‐‐
No education		0.22		5.27	0.076	2.70
Poor wealth index		0.12		2.46	0.014*	1.12
Being youth (15–24 year)		-0.22		-5.60	0.785	1.91
Being divorced and widowed		0.17		5.20	0.001*	2.53
Reside in metropolis region		-0.01		-0.46	0.645	1.64
Having more than one sexual partner		0.18		7.39	0.000*	1.69
Ever not heard about STI		-0.06		-1.56	0.119	1.30
First sexual experience <15 years		0.14		2.11	0.035*	1.96
**Ordinary least square regression diagnostics**						
Number of Observations	620		AIC			3242.132
Multiple R-Squared	0.052		Adjusted R-Squared			0.081
Joint F-Statistic	39.502		Prob(>F), (10,609) DF			0.000
Joint Wald Statistic	268.486		Prob(>chi-squared), (10) DF			0.000
Koenker (BP) Statistic	128.668		Prob(>chi-squared), (10) DF			0.041
Jarque-Bera Statistic	31.315		Prob(>chi-squared), (2) DF			0.182

BP = Bruesch-Pagan, AIC = Akaike information criterion, DF = degrees of freedom

### Geographically weighted regression analysis

Model comparison was done by comparing AIC and R-squared values for each model. A model with a small AIC and a high R^2^ value was considered the best model. Thus, the MGWR model was found to be favorable with AIC and R^2^ values of 1327.661 and 0.151, respectively. Poor wealth index, being divorced and widowed, having more than one sexual partner, and having an early first sexual experience (<15 years) were found to be positively related to HIV seropositivity **(Table 4)**.

**Table 4:** Summary of geographically weighted regression analysis result and model comparisons.

Variables	Mean	SD	Minimum	Median	Maximum
Intercept	-0.109	0.14	-0.32	-0.03	0.24
Poor wealth index	0.181	0.48	0.15	0.21	0.45
Being divorced and widowed	0.173	0.04	0.12	0.21	0.28
Having more than one sexual partner	0.190	0.13	0.08	0.12	0.26
First sexual experience <15 years	0.170	0.23	0.04	0.19	0.41
**Model comparison (OLS vs. MGWR)**					
Parameters	OLS		MGWR		
AIC	3242.132		1327.661		
R-Squared	0.052		0.151		
Adjusted R-Squared	0.081		0.218		

**Fig 7:**
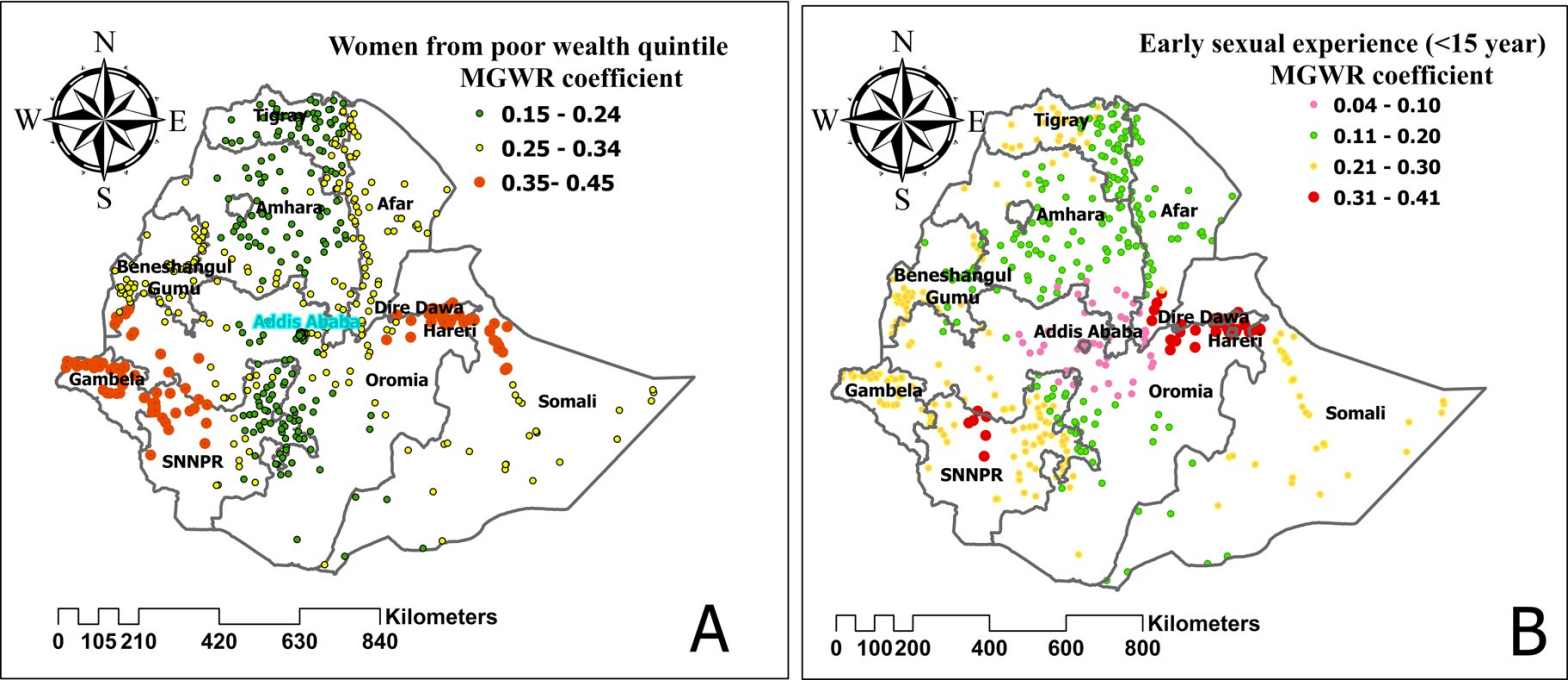
The spatial mapping of geographically weighted regression coefficients by poor wealth index (A) and early sexual experience (B) to predict the hotspot of HIV seropositivity among women in Ethiopia.

Furthermore, the MGWR graph shows that any increase in the number of women in poor wealth index categories increases the prevalence of HIV seropositivity in Gambela, Harari, and Dire Dawa. Additionally, with the increase in the number of women experiencing early sexual intercourse, the incidence of HIV also increases, particularly in Harari and Dire Dawa **(Fig 7)**.

## Discussion

This study aimed to determine the spatial pattern of HIV seropositivity among women population and its predictors. Significant hotspot clustering of HIV seropositivity was detected in Addis Ababa, Harari, Dire Dawa, and Gambela regions. Poor wealth index, being divorced and widowed, having more than one sexual partner, and having early first sexual experience <15 years were found significantly associated factors of HIV seropositivity.

This study revealed that HIV seropositivity among women in Ethiopia varies geographically, from 0.02% to 6.16%. This finding is supported by another study conducted in Zimbabwe [35], South Africa [36] and Malawi [37]. The observed spatial dependency of HIV seropositivity can be attributed to the influence of sociodemographic factors within the population. Geographical variations in factors such as age distribution, residential patterns, educational attainment, marital status, employment status, and socioeconomic status have been extensively documented to be strongly correlated with HIV prevalence across various regions [38–40]. The above findings underscore the need for tailored policy and program interventions. Targeted intervention programs should be implemented in regions with higher prevalence rates to address specific needs, including increased access to testing, education, and support services.

Significant hotspot clustering of HIV seropositivity was detected in the Addis Ababa, Harari, Dire Dawa, and Gambela region. The high clustering of HIV among women in metropolis areas (Addis Ababa, Harari, and Dire Dawa) could be due to an increased population mobility and an increase in sexual partnerships within urban areas. Urban settings often facilitate more frequent interactions and exchanges, potentially increasing the risk of exposure to the virus. Moreover, factors such as population density and social networks conducive to multiple partnerships contribute to elevated sexual activity, further increasing the likelihood of HIV transmission. Socio-economic disparities in metropolitan areas [41]. In addition, commercial sex workers, who are greatest contributors to for HIV prevalence (18.7%), are more common in metropolis areas [23]. Furthermore, the Gambela region was also a high-hotspot area of HIV prevalence. A recent study conducted in the Gambela region revealed that the sociocultural factor known as "Tifo Bet," which translates to "male youth seeking independence from their parents," is responsible for the high prevalence of HIV. This could further create the opportunity to engage in multiple unsafe sexual practices with local young girls [42].

A poor wealth index was found to be a predictor of HIV seropositivity among women in hotspot areas. This finding is in a row with previous studies [43, 44]. Women in metropolis areas with lower socioeconomic status are more likely to engage in commercial sex workers for the sake of sources of income, which increases the risk of HIV seropositivity. This finding highlights the need for special attention to women to engage in different sectors to attain economic independence. Policy and program implications include implementing economic empowerment programs tailored to these women, enhancing access to healthcare services, integrating poverty alleviation with HIV prevention efforts and designing gender-sensitive programs. Addressing socioeconomic disparities in HIV prevalence among women in hotspot areas requires multifaceted approaches that prioritize empowerment, access to healthcare, and gender equality. Being divorced and widowed increases HIV seropositivity in hotspot areas. Similarly, previous studies reported similar findings [45, 46]. This might be related to the presumption that marriage restricts the number of sexual partners, thereby offering protection against HIV. Addressing the unique vulnerabilities faced by divorced and widowed individuals, such as social isolation and economic insecurity, is paramount in HIV prevention efforts.

Having more than one sexual partner is also a contributing factor to an increase in HIV seropositivity among women in high hotspot areas (Addis Ababa, Harari, and Dire Dawa). This finding is in line with the previous studies conducted in South Africa [36], Malawi [47] and Kenya [48]. This is because each new sexual partner represents a new opportunity to be exposed to an STI. The more sexual partners a woman has, the greater the likelihood that one of those partners will be infected with an STI. The findings suggest that addressing the issue of having multiple sexual partners is crucial in combating the increase in HIV seropositivity among women in high hotspot areas. Policy and program implications could include targeted education and awareness campaigns focusing on promoting safer sexual practices,and accessible and affordable services for HIV testing, counseling, and treatment. The earlier the age at first sexual experience, the greater the risk of HIV seropositivity in Hotspot areas. This result is consistent with other results [12, 49–51]. Delaying sexual debut is associated with increased condom use at first intercourse [52]. Furthermore, early coital debut is unprotected, leading to HIV transmission. Early sexual activity is a sign that health promotion campaigns are needed to raise awareness of individual self-care to prevent HIV. The findings indicating a correlation between earlier age at first sexual experience and increased risk of HIV seropositivity in hotspot areas have significant policy and program implications. First, there is a need for targeted sexual education programs aimed at adolescents and young adults residing in hotspot areas, emphasizing the importance of delaying sexual debut and promoting safer sexual practices. Additionally, efforts should be made to increase accessibility to HIV testing and counseling services in these areas, particularly with a focus on reaching younger populations.

This study uses nationally representative data. Thus, it can be generalizable for all women at the national level and has a better statistical power. In addition, this study uses SaTScan and spatial distribution analysis, which are vital for identifying eographically specific information. The secondary nature of the data hider to explore further predictors of HIV seropositivity. Since the data were cross-sectional, it is difficult to draw a causality relationship between the independent and outcome variables.

## Conclusion and recommendations

In Ethiopia, significant spatial clustering of HIV seropositivity among women was found. Significant hotspots were identified in eastern Ethiopia (Harari and Dire Dawa), the capital city (Addis Ababa), and southwestern Ethiopia (Gambela Region). The distribution of HIV seropositivity was not random. Poor wealth index, being divorced or widowed, having more than one sexual partner, and having first sexual experience <15 years were found to be predictors of geographical variation of HIV seropositivity among women. Thus, program planners and policymakers should develop programs encouraging early detection and initiation of antiretroviral therapy (ART) in hotspot areas, which are highly recommended. Governmental and non-governmental entities play a pivotal role in fostering economic empowerment for women. This endeavor is crucial as it grants women greater autonomy over their lives, reducing their vulnerability to resorting to precarious activities for survival, such as risky sexual behavior, thereby reducing the transmission of HIV. Public health institutes and health organizations should disseminate reproductive health education towards limiting the number of sexual partners, and having protected sex is essential. Formulating marriage counseling and support services could play a significant role in reducing the likelihood of divorce and contributing to the decrease in HIV seropositivity among women. Furthermore, the ministry of health and community-based organizations should develop comprehensive sex education programs in schools and community settings regarding the consequences of early first sexual debuts, which might play a role in reducing HIV seropositivity in women. Future prospective research addressing factors that contribute to spatial variation in HIV seropositivity is recommended for researchers.
